# Incoordination during the pharyngeal phase in severe dysphagia due to lateral medullary syndrome

**DOI:** 10.1002/ccr3.3890

**Published:** 2021-02-09

**Authors:** Kenjiro Kunieda, Takafumi Sugi, Tomohisa Ohno, Akiko Nomoto, Takashi Shigematsu, Hideaki Kanazawa, Ichiro Fujishima

**Affiliations:** ^1^ Department of Neurology Gifu University Graduate School of Medicine Gifu Japan; ^2^ Department of Rehabilitation Medicine Hamamatsu City Rehabilitation Hospital Hamamatsu Japan; ^3^ Department of Dentistry Hamamatsu City Rehabilitation Hospital Hamamatsu Japan

**Keywords:** central pattern generator, high‐resolution manometry, incoordination, lateral medullary syndrome, upper esophageal sphincter

## Abstract

One of the mechanisms of severe dysphagia due to lateral medullary syndrome may be a reversed pressure gradient caused by incoordination of pharyngeal contractility and UES opening during swallowing.

## Graphical abstract

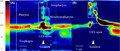

## 1 INTRODUCTION

Lateral medullary syndrome (LMS) is a neurological disease caused by a lesion in the lateral part of the medulla oblongata.^1^ Dysphagia, a common complication of LMS, is clinically important because of its associations with aspiration pneumonia, malnutrition, increased mortality, and decreased quality of life. The swallowing center, which includes the nucleus ambiguus (NA) and the nucleus tractus solitarius (NTS), coordinates the pharyngeal phases of swallowing. Fujishima et al^2^ reported upper esophageal sphincter (UES) and pharyngeal incoordination during swallowing in patients with dysphagia with LMS using a conventional sensor. Surface electromyography and needle electromyography are also useful methods to assess the muscle activity of the cricopharyngeal muscle during swallowing. However, the pathophysiology of dysphagia has not been fully elucidated in these patietns.^1^, ^2^

We present a case wherein high‐resolution manometry (HRM) was used to examine the pathophysiology of a patient with LMS‐related severe bulbar dysphagia.

## 2 CASE REPORT

**FIGURE 1:**
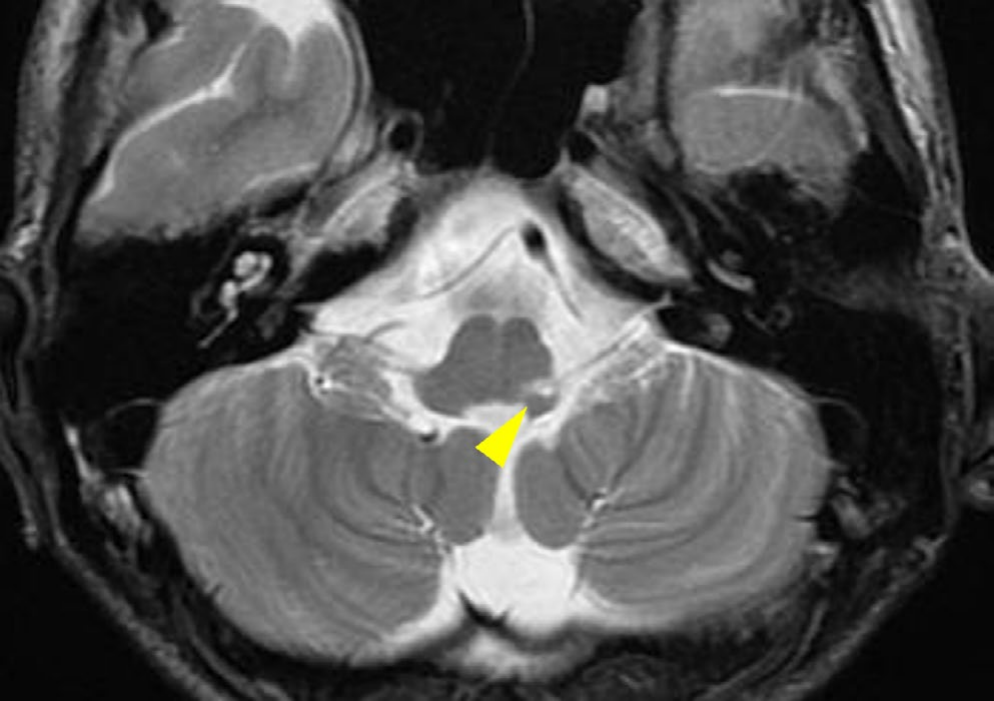
Magnetic resonance imaging 14 mo after onset. T2‐weighted image showing high intensity and confined to a small region of the left dorsolateral medulla (yellow arrow)

A 41‐year‐old man presented with gait disorder, hoarseness, and dysphagia. He was diagnosed with left LMS due to infarction (Figure 1), and oral antiplatelet therapy was initiated. His neurological deficit almost improved except for severe dysphagia. Eleven months after onset, he underwent percutaneous endoscopic gastrostomy. Fourteen months after onset, he was transferred to the rehabilitation department of our hospital for dysphagia treatment.

**FIGURE 2:**
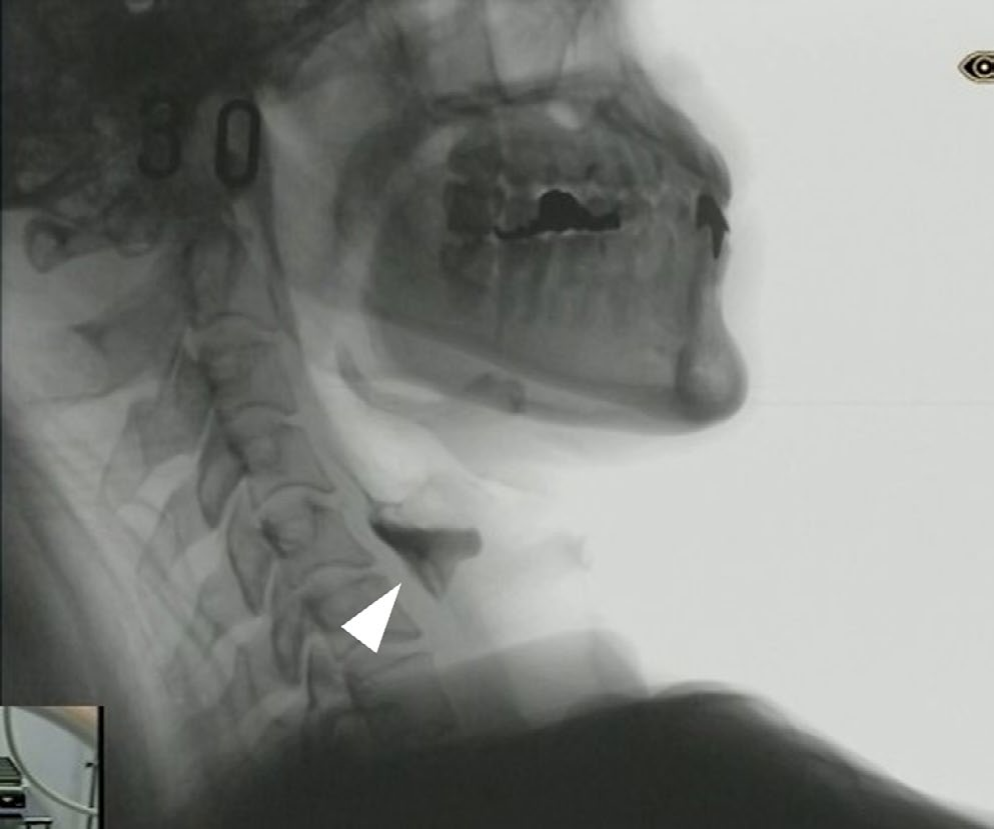
Videofluoroscopic examination of swallowing 14 mo after onset. Presence of a severe bolus residue in the pyriform sinus owing to weak pharyngeal contraction and an impaired UES relaxation (arrowhead)

**FIGURE 3:**
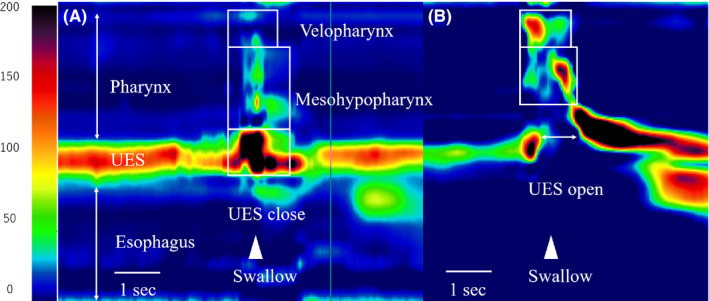
Pressure topographies of the patient (A) and of a normal individual (B). Spatiotemporal plots of dry swallows. Y‐axis, catheter position; X‐axis, time. Pressure is indicated using the color scale. Maximum pharyngeal pressures measured at the velopharynx and meso‐hypo pharynx. UES relaxation duration and UES pressure during swallowing are evaluated. UES, upper esophageal sphincter

Videoendoscopic examination of swallowing revealed fixed left vocal cord in the midline position, insufficient left nasopharyngeal contraction, pooling of saliva in the left pyriform sinus, and weakened left pharyngeal contraction. Videofluoroscopic examination of swallowing revealed weak pharyngeal contractility and impaired UES function. The bolus remained in the pyriform sinus and did not pass through the pharynx (Figure 2). The swallowing pressure along the pharynx and UES was measured using HRM (Unisensor AG). HRM revealed weak pharyngeal constriction and strong constriction of the UES muscle zone (Figure 3). We used the pharyngeal contractile integral (CI; mmHg‐cm‐s) to evaluate pharyngeal swallowing pressure.^3^, ^4^ Five dry swallows were examined during which the patient's velopharyngeal CI (VPCI) and mesohypopharyngeal CI (MHPCI) values were 4.1 ± 2.9 and 72.0 ± 16.2, respectively (as reference values for these data, the VPCI and MHPCI values for 3‐mL thickened liquids in our previous report were 124.3 ± 50.3 and 193.2 ± 34.1, respectively^4^). Regarding the patient's UES function, the UESCI value was 713.7 ± 72.7, indicating strong contraction during swallowing.

The patient received nutritional treatment, respiratory training, and swallowing rehabilitation (ie, balloon catheter dilatation, Shaker exercise, and vacuum swallowing therapies). He was discharged 16 months after the initial onset. He currently continues the swallowing rehabilitation at home and receives treatment on an outpatient basis. This study was approved by the hospital's ethics committee.

## 3 DISCUSSION

To the best of our knowledge, this is the first report to biomechanically clarify the pathophysiology of severe dysphagia due to LMS using HRM. The main finding of this study is that the UES closed strongly during swallowing in a patient with bulbar dysphagia with LMS. The UES remains tonically contracted between swallows. During normal swallowing, the UES opens, and the bolus passes from the pharynx to the upper esophagus, and the UES pressure drops to zero or below; however, the UES pressure in our patient increased abnormally during swallowing.

Notably, we calculated the pharyngeal CI as a measure of contractility to evaluate the pressure gradient between pharyngeal contraction and impaired UES function. The CI was calculated as amplitude × duration × length of muscular contraction ≥20 mm Hg. It can act as a useful indicator of pharyngeal swallowing disorders.^2^ The CI is a measure of the “vigor” of contractility and has the potential to provide objective representation of pharyngeal and UES muscular functions. Compared with normal individuals, this patient's VPCI and MHPCI values were low owing to pharyngeal contractile weakness, whereas, UESCI was remarkably high owing to abnormal UES closure. The weakened pharyngeal contraction and the remarkable UES closure during swallowing significantly interfere with the pharyngeal passage of a bolus in this patient.

High‐resolution manometry provided a more accurate and quantitative assessment of swallowing function. The high‐pressure zone of the UES is narrow and asymmetric, and it moves up and down with the elevation of the larynx during swallowing. Therefore, a catheter with circumferential sensors helps evaluate the UES function precisely. Decreased pharyngeal contraction and elevation of UES pressure during swallowing were reported using conventional sensors in previous studies^2^, ^5^ that found it difficult to precisely measure the “vigor” of abnormal UES contractility. Furthermore, the change in UES pressure with time is also characteristic. In normal individuals, the UES pressure increased from the resting pressure before and after swallowing. Contrarily, in the patient, the UES pressure decreased from the resting pressure before and after swallowing.

The central pattern generator (CPG) for swallowing, located in the medulla, controls the oropharyngeal phase of the swallowing sequence.^1^ Abnormalities in the UES function owing to dysphagia‐causing LMS have been reported, but there are a few reports regarding the associated pathophysiology. NA and NTS, located in the lateral medulla of the CPG, control the muscles of the pharynx and UES. Therefore, an LMS lesion that includes the NA and NTS can cause weak pharyngeal contraction and abnormal UES relaxation. These abnormal findings are significant on the side ipsilateral to the affected side of the medulla. The swallowing reflex is often absent. A strongly closed UES during swallowing might reflect the high spasticity of the cricopharyngeal muscles.

Continuous swallowing rehabilitation (ie, balloon catheter dilatation and Shaker exercise), nutritional treatment, and respiratory muscle training would be beneficial for these patients with failed UES relaxation, as they facilitate opening of the UES. The swallowing rehabilitation techniques including balloon catheter dilatation and Shaker exercise aim at facilitating the opening of the UES. If there is no sufficient improvement with these approaches, then cricopharyngeal myotomy can be considered to improve the passage of the bolus from the pharynx to the upper esophagus. This is the only case report on this topic, which may be a limitation. Therefore, future studies should evaluate additional cases to verify our case findings.

## 4 CONCLUSION

One mechanism of severe dysphagia due to lesions in the medulla may be the reversed pressure gradient caused by the incoordination of weakened pharyngeal contraction and the remarkable UES closure. Further studies should elucidate the pathophysiology, prognosis, and treatment of severe dysphagia due to LMS.

## CONFLICT OF INTEREST

The authors state that they have no conflicts of interest.

## AUTHOR CONTRIBUTIONS

KK, TS, HK, and IF: performed study concept and design. KK, TS, and AN: acquired the data. KK, TO, AN, and IF: analyzed and interpreted data. KK, TS, TO, AN, TS, HK, and IF: drafted the manuscript.

## ETHICAL APPROVAL

This study was approved by the hospital's ethics committee.

## INFORMED CONSENT

This study was approved by the Ethics Committee of our hospital. The patient provided informed consent.

## Data Availability

Data available on request from the authors.
